# Sun Baths

**Published:** 1882-09

**Authors:** 


### SUN-BATHS

Cost nothing, and are the most refreshing, life-giving baths that one can take, whether sick or well. Every housekeeper knows the necessity of giving her woolens the benefit of the sun, from time to time, and especially after a long rainy season or a long absence of the sun. Many will think of the injury their clothes are liable to from dampness, who will never reflect, that an occasional exposure of their own bodies to the sun-light is equally necessary to their own health.

The sun-baths cost nothing and that is a misfortune, for people are still deluded with the idea that those things only can be good or useful which cost money, and they will cheerfully pay away their dollars for Turkish and Russian Baths, when they could get any number of sun-baths, which would be far more beneficial to them, for nothing.

Let it not be forgotten, that three of God's most beneficent gifts to man—three things the most necessary to good health—sun-light, fresh air and water are *free* to all; you can have them in abundance, without money and without price, if you will. If you would enjoy good health, then see to it, that you are supplied with pure air to breathe all the time; that you bathe for an hour or so every day in the sun-light; and that you quench your thirst with no other fluid than water.

In regard to sun-baths, let any invalid, who reads this and who has been housed for some time, take an occasional walk in the sun, if it should be only on the piazza, and observe the effect. In our opinion, he will find it the most healthful bath he has ever taken.

Sleeping-rooms should be selected in such parts of the house as have the most benefit from the rays of the sun; the bed and bed-clothes should be thoroughly aired and kept in the sun as long as possible every day. Many of the sleeping-rooms in our hotels are so situated as never to feel the influence of the sun's rays, and those who occupy such rooms for any length of time, are simply committing suicide. We have in mind, now, a large hotel in the vicinity of New York City, where not less than two hundred persons are usually located for the winter, in which a large proportion of the bed-rooms are in the centre of the building, into which the sun-light never penetrates. As a corollary, the doctors' gigs are seen standing before the house at all hours of the day.

The Italians have a proverb, which says, "*Dove non entrar il sole deve andar il medico*;"* and with them, the first point to be considered in the selection of a house, is: *What is its exposure to the sun?* and they are careful to locate their sleeping-rooms on the side of the house where there will be the most sun.

Again, too many houses in most of our cities, and very many in country villages, are completely buried from the sun by shade trees. Elegant establishments, these houses, whose occupants can command every luxury, within the reach of wealth; saloons into which rank, beauty and fashion are welcomed, but from which the sun-light of Heaven is totally excluded by shade trees!

At the risk of being denounced as an iconoclast, we would lay the axe to the root of at least two-thirds of the shade trees which surround our houses and line our streets.

* Where the sun does not enter the doctor must.

